# Alterations in IAA, ABA and Polyamine Pool Levels of Two Winter Wheat Cultivars Caused by Drought Stress—Interactions with Melatonin

**DOI:** 10.3390/plants15203225

**Published:** 2026-10-21

**Authors:** Zornitsa Katerova, Tsvetina Nikolova, Irina I. Vaseva, Dessislava Todorova, Iskren Sergiev

**Affiliations:** Institute of Plant Physiology and Genetics, Bulgarian Academy of Sciences, Acad. Georgi Bonchev Street, Bldg. 21, 1113 Sofia, Bulgaria; zornitsa@bio21.bas.bg (Z.K.); tnikolova00@bio21.bas.bg (T.N.); vaseva@bio21.bas.bg (I.I.V.); iskren@bio21.bas.bg (I.S.)

**Keywords:** ABA, IAA, melatonin, polyamine fractions, priming, wheat, soil drought

## Abstract

The present study investigates the potential of the plant growth regulator melatonin to alleviate the effects of moderate soil drought on two Bulgarian wheat cultivars (drought-tolerant and less-drought-tolerant) by modulating indole-3-acetic acid (IAA), abscisic acid (ABA) and polyamine (PA) levels. Drought induced ABA and decreased IAA content, while a specific response reflecting wheat tolerance was observed in the levels of the polyamines spermine, spermidine and putrescine. The less-drought-tolerant cultivar Fermer accumulated supernatant-conjugated PAs, while in the drought-tolerant cultivar Gines, the pellet-bound fraction increased. After four days of recovery, the decreased levels of IAA and pellet-bound fractions of PAs, along with the still-increased ABA content in cv. Fermer implied drought stress had a residual effect. In total, 75 µM melatonin (added to the irrigation water 24 h before or after drought) alleviated drought-induced alterations in IAA and ABA, with a more pronounced effect in cv. Fermer. The less-drought-tolerant variety responded to drought through a melatonin-modulated reorganization and accumulation of PAs, particularly in their conjugated forms, whereas cv. Gines maintained a comparatively stable polyamine pool with smaller treatment-induced changes. Overall data indicated that the outcome of melatonin treatment on the levels of IAA, ABA and the polyamine pool under drought is rather complex and depends on the specificity of the cultivars’ tolerance and the timing of melatonin application.

## 1. Introduction

Water deficiency is a common stress factor worldwide, affecting a variety of crops in an increasing number of agricultural regions. Wheat is an extensively grown staple crop. Although it is relatively tolerant to water shortages, prolonged periods of water scarcity, especially in its early development stages, have negative effects on every aspect of plant physiology and ultimately compromise wheat growth, quality and yield. All of these factors make drought a substantial problem that needs scientifically based solutions. An ecologically friendly approach focused on lessening the negative drought stress effects in plants is the so-called priming method [1,2,3]. Priming can be accomplished through preliminary exposure to mild stress or by pre-application of different metabolically active compounds. Pre-treatment could be performed on seeds, seedlings, mature plants, and also on leaves or roots. The priming agents were classified into the following groups: chemical compounds, biostimulants and nanomaterials [4]. Biostimulant formulations contain naturally derived compounds and can be classified into different categories based on their main components, such as humic substances, seaweed extracts, hydrolyzed proteins and amino acid-containing products, microorganisms, etc. Usually, the positive effects of biostimulants do not depend on a single compound [2,5,6,7,8]. Many biostimulants contain phytohormones and/or plant growth regulators and their application is categorized as so-called hormonal priming. In wheat, hormonal priming is among the common strategies reported to mitigate the consequences of drought stress [3]. Moreover, hormonal priming is recognized to promote a variety of consecutive biochemical and molecular modifications, which activate stress-responsive genes, lead to accumulation of defense compounds, and modify phytohormonal signaling, ending with enhanced and effective protection in plants [9,10,11]. The primed plants are equipped with improved stress tolerance that lasts for an extended period due to the so-called “stress memory”—the effect, which is noted to be epigenetically based and associated with hormone signal transduction, ROS scavenging, etc. [10,12]. In addition, biostimulants can be applied not only before stress, but also during or after stress.

Melatonin (N-acetyl-5-methoxytryptamine, Mel) is a natural indole compound present in animals, plants, fungi and microorganisms. Together with serotonin, dopamine, acetylcholine and gamma-aminobutyric acid, melatonin represents an animal neurotransmitter [9], which, in plants, interacts with phytohormones and has recently been acknowledged as a master regulator improving plant physiological responses to a variety of stresses [13,14,15,16]. Evidence has accumulated for the positive role of melatonin pre-treatment on various plants subjected to different environmental constraints, including drought [15,17,18,19,20,21,22,23,24,25,26]. Melatonin applied as an exogenous biostimulator has been shown to render beneficial effects under abiotic stress [27,28] and appears to be a powerful tool to withstand various environmental constraints in plants [29,30]. Melatonin has gained attention as a potent plant protector and an important ingredient in biostimulant formulations, exerting anti-senescence and anti-stress effects [14,20,31,32,33,34,35,36]. The major positive effects of melatonin on plants subjected to drought were revealed to be increased antioxidant capacity, improved growth, photosynthesis and water retention [18,25,37,38,39,40,41,42,43,44]. Its key role in signaling pathways, including polyamines (PAs), abscisic acid (ABA) and indole-3-acetic acid (IAA), and in mediating the cross-talk with other phytohormones, is another important advantage of melatonin [25,32,33,38,39,40,41,42,45,46]. In fact, many plant growth regulators such as abscisic acid, auxins, ethylene, cytokinins, polyamines, salicylic acid, jasmonic acid, gibberellins, brassinosteroids, and strigolactones are significant contributors to drought acclimatization [47,48,49], and their cross-talk plays a vital role in the survival of stressed plants. It was reported that among six melatonin-pathway intermediates (tryptophan, tryptamine, serotonin, 5-methoxy tryptamine, N-acetyl serotonin, and melatonin), tryptophan is the most abundant metabolite in wheat [50]. Both tryptophan derivatives, melatonin and the auxin IAA, are shown to be important growth regulators that participate in plant growth promotion and adaptation to various stress factors, such as drought, with concentration-dependent outcomes [51,52,53]. Although the melatonin-induced primary root growth in Arabidopsis is IAA-dependent [53], their interactions remain disputable. Zhang et al. [52] suggested that melatonin signaling may play a key role in responses under stress, and IAA signaling could be significant in plant growth and development.

Polyamines are another group of plant growth regulators with a significant effect on plants grown under stress, including drought [54,55,56,57,58]. They belong to aliphatic compounds with two or more positively charged amino groups and are known as plant growth regulators with compatible solutes and antioxidant properties [59,60]. Due to their polycationic nature, they can interact with negatively charged macromolecules such as nucleic acids, proteins and membrane phospholipids. The most common PAs in plants are putrescine (Put), spermidine (Spd) and spermine (Spm), which possess two, three and four amino groups respectively; they present as free and conjugated with low-molecular-weight compounds (SN-conjugated), and are bound to macromolecule (pellet-bound) fractions. In general, the PA pool was observed to fluctuate under various stresses, including drought, and is dependent on different factors such as stress duration, plant organ, developmental stage, and plant species and genotype [20,38,57,61,62,63]. Polyamines were identified as significant contributors to water stress tolerance [56,57,61,63]. Using *Lotus tenuis* plants, Espasantin et al. [56] proved that the enhanced Put content improved the water balance of cells and controlled ABA levels through altered ABA biosynthesis at the transcriptional level under drought conditions. It was reported that ABA modified PA metabolism by increasing the transcription of biosynthetic genes, inducing Put accumulation, but decreasing Spd and Spm contents in wheat leaves grown under PEG conditions [61]. ABA is a major stress phytohormone in drought acclimation, evoking expression of various stress-related genes [59,61] and participating in stomatal closure, preventing water loss [64,65,66]. The interaction between ABA and auxin is essential for the regulation and adjustment of the stress response, as well as plant development under drought. [67,68]. Furthermore, wheat genotypes vary in their capacity to synthesize ABA during drought conditions [69]. However, the most widely reported result of melatonin application to prevent negative drought stress effects related to ABA is the decreased concentration of this stress hormone, which may cause partial stomatal reopening in barley [19], mung bean [37], tobacco [15], maize [70], soybean [21], and *Malus* species [40].

Recent data have emerged regarding the influence of melatonin on the endogenous levels of numerous plant growth regulators in a number of plants, including tobacco [15], barley [19], mungbean [37], tomato [71,72], soybean [21,22,23,41], *Malus* sp. [40], millet [73] and wheat [20,25,64,74], under drought or osmotic stress. Limited investigations on the content of plant growth regulators after melatonin root application in wheat are based on a PEG-induced drought experimental setup [64,74]. However, closer to the naturally occurring water deficiency is the soil-based experimental model [75,76]. To our knowledge, no information on the effects of melatonin root application is available in the scientific literature, either before stress as a priming agent or after soil drought conditions as a post-stress recovery regulator, on IAA, ABA and PA content in wheat. In previous research [77,78,79], we investigated the effects of melatonin root application before or after soil drought stress on growth, selected oxidative stress markers and non-enzymatic antioxidants, gene expression of proline-related biosynthetic and antioxidant enzymes, and parameters associated with photosynthesis in two Bulgarian wheat varieties with different tolerances—cv. Gines as the drought-tolerant and cv. Fermer as the less-drought-tolerant wheat variety [80,81]. We found that melatonin application differentially influenced the components of antioxidant defense, photosynthesis, and transcript profiling of antioxidant defense in both cultivars depending on their tolerance to drought and time of application (before stress or in the early stages of recovery). This study investigates the effects of exogenous melatonin applied before and after drought on the content of IAA, ABA and different polyamine fractions (free, SN-conjugated and pellet-bound fractions) in two wheat cultivars, expanding our previous findings in this model system.

## 2. Results

### 2.1. Effects of Melatonin and Drought on Endogenous IAA and ABA

**Figure 1:**
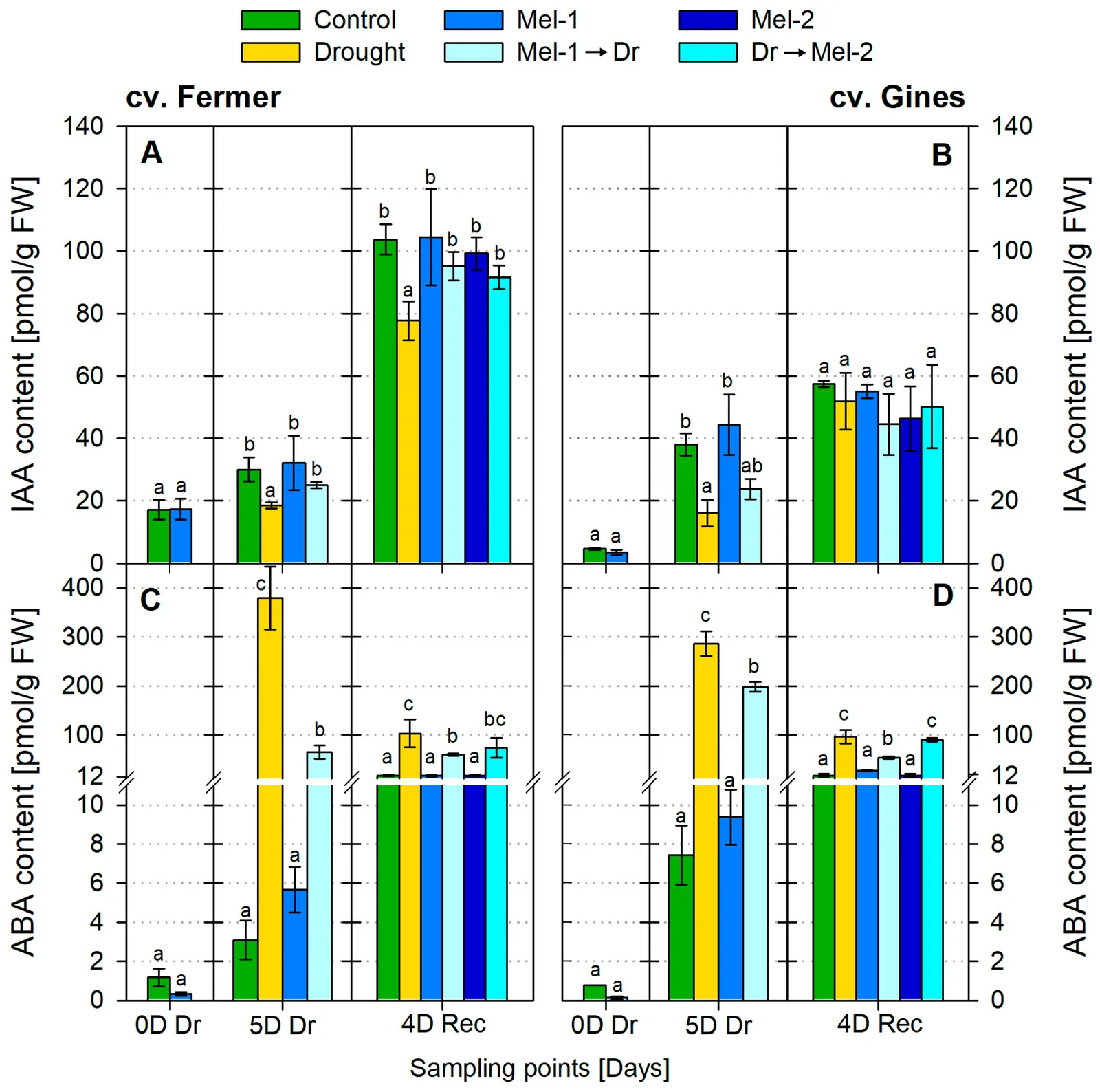
Content of IAA (**A**,**B**) and ABA (**C**,**D**) in the leaves of wheat varieties of Fermer (**A**,**C**) and Gines (**B**,**D**) before drought (0D Dr), at the fifth day of drought (5D Dr) and four days into recovery (4D Rec). The abbreviations within the legends denote the following—Control: normally irrigated or non-treated; Drought: 5 days of water deficiency; Mel-1: melatonin control before drought; Mel-2: melatonin control after drought; Mel-1→Dr: melatonin pre-treatment before drought; Dr→Mel-2: melatonin post-treatment after drought. Error bars designate standard errors (*n* = 6). Different small letters indicate statistically significant differences between treatments for each time-sampling group at *p* < 0.05.

At the end of the stress period (5D Dr), drought significantly decreased the levels of IAA in both wheat varieties compared to the respective controls (Figure 1A,B). After four days of recovery (4D Rec), the IAA values in cv. Fermer (Figure 1A) were still lower, but in cv. Gines (Figure 1B), they reached the control level. Melatonin treatments alone (Mel-1 and Mel-2) did not alter IAA content compared to the respective control values in both cultivars. The combined application of melatonin and drought (Mel-1→Dr and Dr→Mel-2) maintained IAA levels higher than those of the stressed plants and neared the control values in cv. Fermer. However, the IAA content in cv. Gines in plants subjected to the combined application of melatonin before drought (Mel-1→Dr) was lower than the control at 5D Dr. Later, at 4D Rec, IAA content was not significantly different from the control in all variants (Drought, Mel-1→Dr, Dr→Mel-2) of cv. Gines (Figure 1B).

Severely increased ABA content was registered after five days of drought: 120-fold (from 3 to 379 pmol/gFW) higher in cv. Fermer (Figure 1C) and 40-fold (from 7 to 287 pmol/gFW) higher than the respective control in cv. Gines (Figure 1D). This tendency weakened at 4D Rec, and a smaller rise compared to the control was detected in both cultivars. Treatments with melatonin alone did not alter the ABA content compared to the respective control values. At 5D Dr, the application of melatonin before drought (Mel-1→Dr) considerably decreased the ABA level in cv. Fermer (Figure 1C), while in cv. Gines, ABA was reduced to a lesser degree (Figure 1D) compared to the stressed plants. Later, at 4D Rec, the application of melatonin before drought (Mel-1→Dr) further reduced the ABA content in both cultivars. The measured ABA levels were significantly lower than in stressed plants and closer to the respective control. A similar but weaker tendency after four days of recovery was noted due to the application of melatonin after drought (Dr→Mel-2), although values of ABA content did not significantly differ from those of the stressed plants.

### 2.2. Effects of Melatonin and Drought on Endogenous Polyamines

#### 2.2.1. Changes in Putrescine Fraction Levels

**Figure 2:**
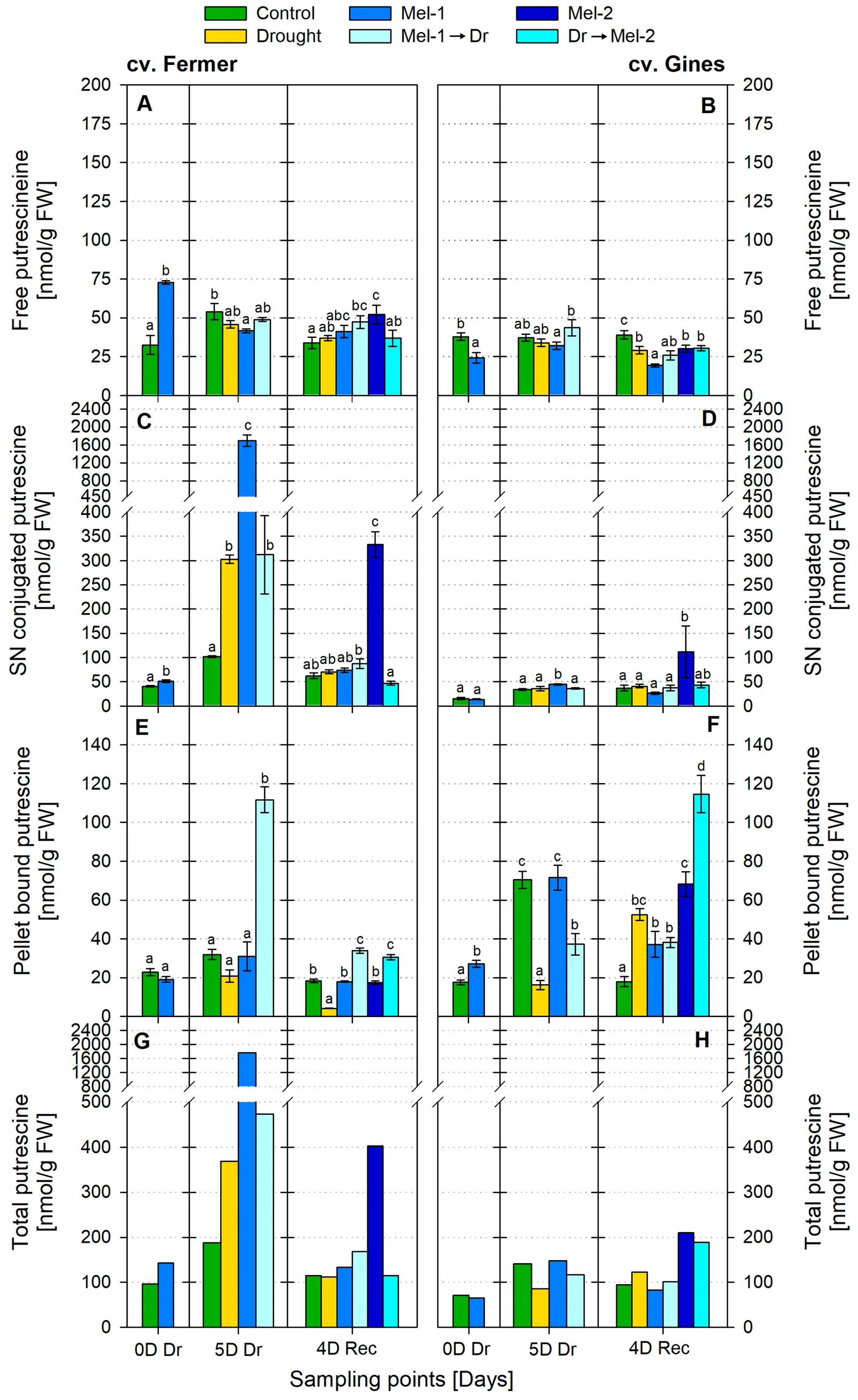
Content of free (**A**,**B**), SN-conjugated (**C**,**D**), pellet-bound (**E**,**F**), and total (**G**,**H**) putrescine in the leaves of the wheat varieties Fermer (**A**,**C**,**E**,**G**) and Gines (**B**,**D**,**F**,**H**) before drought (0D Dr), at the fifth day of drought (5D Dr) and four days into recovery (4D Rec). The abbreviations within the legends denote the following—Control: normally irrigated non-treated; Drought: 5 days of water deficit; Mel-1: melatonin control before drought; Mel-2: melatonin control after drought; Mel-1→Dr: melatonin pre-treatment before drought; Dr→Mel-2: melatonin post-treatment after drought. Error bars designate standard errors (*n* = 6). Different letters indicate statistically significant differences between treatments for each time-sampling group at *p* < 0.05.

Drought stress induced distinct changes in Put accumulation and partitioning in the two cultivars. Free Put showed comparatively slight fluctuations in cv. Fermer (Figure 2A) and in cv. Gines (Figure 2B) after 5D Dr. Later, free Put decreased slightly only in cv. Gines. In cv. Fermer, the most pronounced response was observed in the SN-conjugated fraction, which increased substantially after 5 days of drought and reached values of 303 nmol/gFW (Figure 2C). During early post-drought recovery, the levels tended to decrease and neared those of the control. In contrast, cv. Gines exhibited substantially smaller changes in SN-conjugated Put during the five days of drought and four days of recovery (Figure 2D). The pellet-bound fraction declined after drought in both cultivars (Figure 2E,F), but tended to increase in cv. Gines during recovery and reached 53 nmol/gFW. Total Put (Figure 2G,H) mainly reflected the changes in the SN-conjugated fraction and was substantially increased in cv. Fermer to 370 nmol/gFW.

Initially (0D Dr), pre-treatment with melatonin alone (Mel-1) increased the free Put content in cv. Fermer (Figure 2A, 73 nmol/gFW), while in cv. Gines (Figure 2B), a decrease to 24 nmol/gFW was found compared to the respective controls. After drought and during recovery, no substantial alteration of this fraction was found in both cultivars after Mel-1 alone was combined with drought Mel-1→Dr. However, a significant increase in SN-bound Put was detected in cv. Fermer (Figure 2C) and the level reached 1694 nmol/gFW at 5D Dr after Mel-1 application alone. In contrast, no considerable alterations regarding this fraction were found in cv. Gines (Figure 2D). After four days of recovery the levels tended to decrease and neared the respective controls. Pre-treatment with melatonin (Mel-1→Dr) did not change the SN-conjugated Put in both varieties, and the level was comparable with that of drought-treated plants. The pellet-bound fraction (Figure 2E,F) was not influenced by Mel-1 alone during the entire experimental period in both cultivars, except for a slight increase in cv. Gines after four days of recovery. In combination with drought, melatonin application significantly increased pellet-bound Put in both varieties (especially in cv. Fermer) compared to plants subjected to drought. During early recovery after drought stress, this tendency remained similar but less expressed in cv. Fermer only. Mel-2 was applied alone and in combination with drought (Dr→Mel-2) and had similar effects on Mel-1 but a weaker effect on the SN-conjugated Put fraction, which was better expressed in cv. Fermer (Figure 2C). Pellet-bound Put was additionally increased during recovery after Dr→Mel-2 treatment in cv. Gines (Figure 2F). The total Put pool resulted mainly from the SN-conjugated fraction in both cultivars.

#### 2.2.2. Changes in Spermidine Fraction Levels

**Figure 3:**
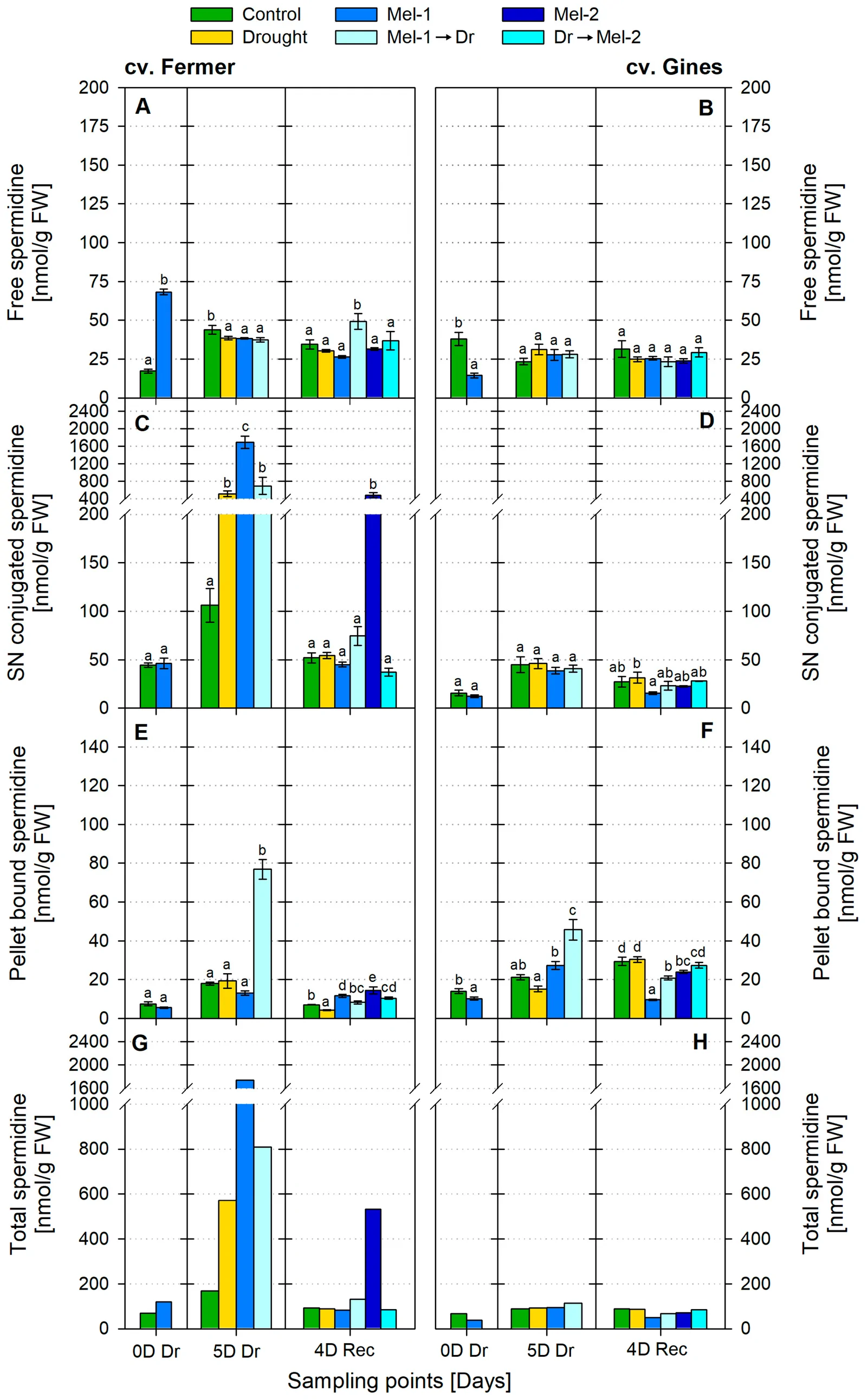
Content of free (**A**,**B**), SN-conjugated (**C**,**D**), pellet-bound (**E**,**F**), and total (**G**,**H**) Spd in the leaves of wheat varieties of Fermer (**A**,**C**,**E**,**G**) and Gines (**B**,**D**,**F**,**H**) before drought (0D Dr), at the fifth day of drought (5D Dr) and four days into recovery (4D Rec). The abbreviations within the legends denote the following—Control: normally irrigated non-treated; Drought: 5 days of water deficit; Mel-1: melatonin control before drought; Mel-2: melatonin control after drought; Mel-1→Dr: melatonin pre-treatment before drought; Dr→Mel-2: melatonin post-treatment after drought. Error bars designate standard errors (*n* = 6). Different letters indicate statistically significant differences between treatments for each time-sampling group at *p* < 0.05.

Drought did not change the content of free Spd in cv. Fermer (Figure 3A). The content of SN-conjugated Spd was substantially increased up to 515 nmol/gFW compared to the control (Figure 3C) on the fifth day of drought, while pellet-bound Spd (Figure 3E) was not significantly altered. As a result, the total Spd fraction was also increased to 572 nmol/gFW after stress (5D Dr) in cv. Fermer (Figure 3G). In cv. Gines, drought did not alter the levels of free (Figure 3B), SN-conjugated (Figure 3D), pellet-bound (Figure 3F) or total (Figure 3H) Spd. Later (4D Rec), no significant changes were observed in both cultivars.

Similarly to Put, application of Mel-1 alone increased free Spd in cv. Fermer (68 nmol/gFW, Figure 3A) and decreased it in cv. Gines (14 nmol/gFW, Figure 3B) compared to the respective controls before drought (0D Dr). In combination with drought, melatonin caused an increase in this fraction during early post-drought recovery only in cv. Fermer. The changes in the SN-conjugated fraction followed the same tendency as Put in both cultivars (Figure 3C,D). Regarding the pellet-bound Spd (Figure 3E,F), at the end of drought, Mel-1→Dr increased the content of this fraction compared to drought-treated plants, with more obvious alterations in cv. Fermer. After four days of recovery, the levels of pellet-bound Spd were closer to the respective controls.

Post-drought treatment with melatonin alone (Mel-2) increased SN-conjugated Spd substantially (487 nmol/gFW) alongside the corresponding total fractions (532 nmol/gFW) in cv. Fermer. During early post-drought recovery, the combined melatonin application after drought (Dr→Mel-2) brought the studied Spd fraction values close to the respective control values in both wheat varieties.

#### 2.2.3. Changes in Spermine Fraction Levels

**Figure 4:**
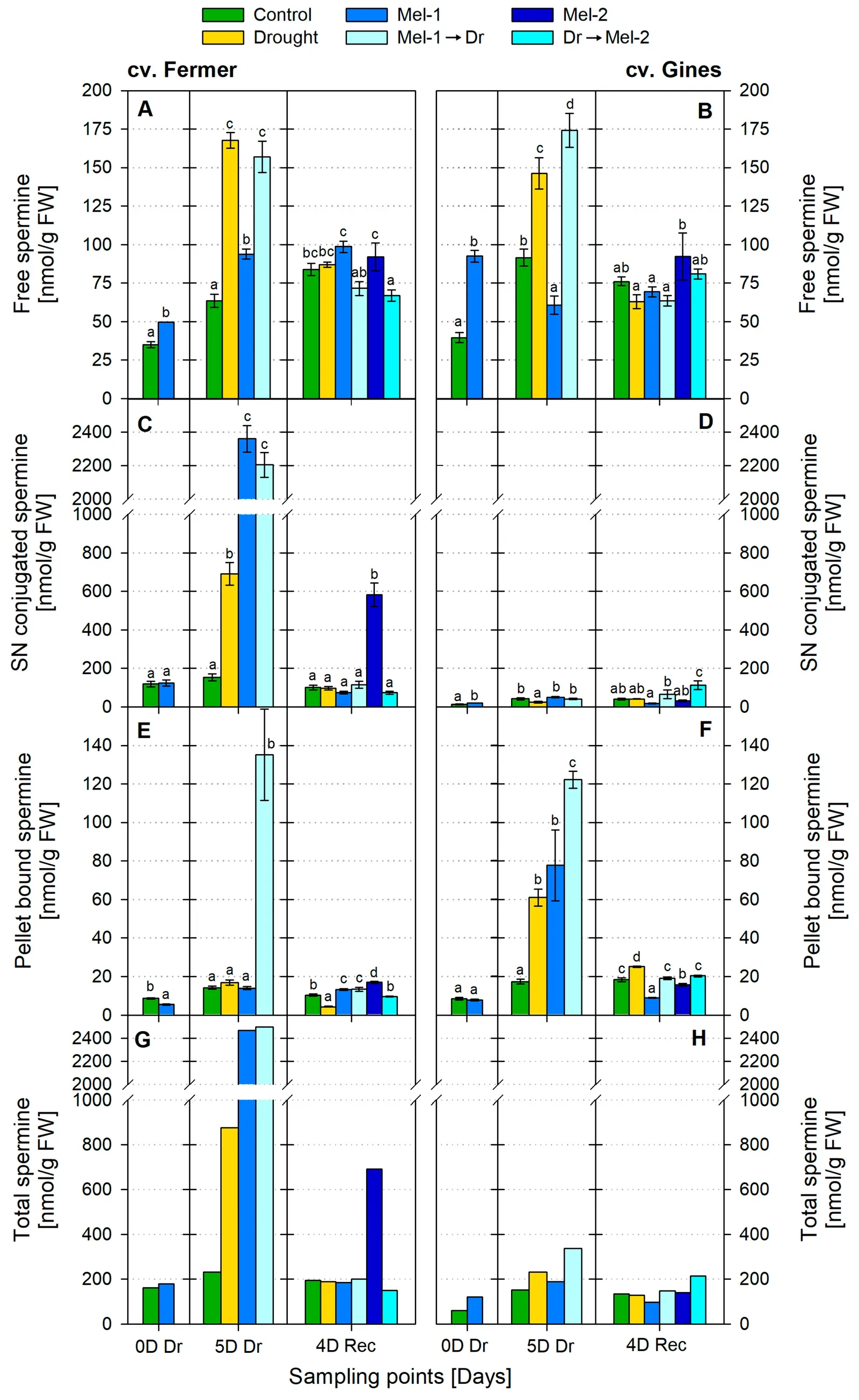
Content of free (**A**,**B**), SN-conjugated (**C**,**D**), pellet-bound (**E**,**F**), and total (**G**,**H**) Spm in the leaves of wheat varieties of Fermer (**A**,**C**,**E**,**G**) and Gines (**B**,**D**,**F**,**H**) before drought (0D Dr), at the fifth day of drought (5D Dr) and four days into recovery (4D Rec). The abbreviations within the legends denote the following—Control: normally irrigated non-treated; Drought: 5 days of water deficit; Mel-1: melatonin control before drought; Mel-2: melatonin control after drought; Mel-1→Dr: melatonin pre-treatment before drought; Dr→Mel-2: melatonin post-treatment after drought. Error bars designate standard errors (*n* = 6). Different letters indicate statistically significant differences between treatments for each time-sampling group at *p* < 0.05.

Drought increased free Spm (Figure 4A,B) in both varieties (168 nmol/gFW in cv. Fermer, and 146 nmol/gFW in cv. Gines) five days after stress (5D DR) compared to the respective control levels.

The content of SN-conjugated Spm was amplified (691 nmol/gFW) in cv. Fermer (Figure 4C), but a small decrease was detected in cv. Gines (Figure 4D) after five days of drought. Pellet-bound Spm slightly decreased in cv. Fermer (Figure 4E, 4D Rec) but increased (61 nmol/gFW at 5D Dr and 25 nmol/gFW at 4D Rec) in cv. Gines (Figure 4F). Total Spm increased substantially in cv. Fermer (876 nmol/gFW, Figure 4G), and slightly in cv. Gines (Figure 4H) after five days of drought.

Melatonin treatment also altered Spm partitioning in similar to Put and Spd manner. In cv. Fermer, Mel-1 strongly enhanced and SN-conjugated Spm accumulation (2360 nmol/gFW) during drought (Figure 4C), which resulted in the increase in the total amount (Figure 4G, 2468 nmol/gFW), whereas Mel-1→Dr produced a substantial increase in the pellet-bound fraction (134 nmol/gFW, Figure 4E). During early recovery phase, Mel-2 alone was associated with persistence of a relatively large SN-conjugated Spm, which is resulting in elevated total Spm pool.

In cv. Gines, melatonin applied before drought (Mel-1→Dr) additionally enhanced both free (173 nmol/gFW, Figure 4B) and pellet-bound (122 nmol/gFW, Figure 4F) Spm as compared to drought-treated plants. However, the SN-conjugated fraction in cv. Gines remained relatively low after Mel-1→Dr (Figure 4D), and consequently total Spm (Figure 4H) did not approach the concentrations observed in cv. Fermer (Figure 4G). After four days of recovery, the content of all Spm fractions in cv. Gines under all treatments remained near the control.

Overall, the data demonstrated pronounced cultivar-specific differences in polyamine fractions, with cv. Fermer showing a stronger drought- and melatonin-associated accumulation of the SN-conjugated form than cv. Gines.

#### 2.2.4. Radar Plot Presentation of the Studied Polyamines Normalized to the Control

**Figure 5:**
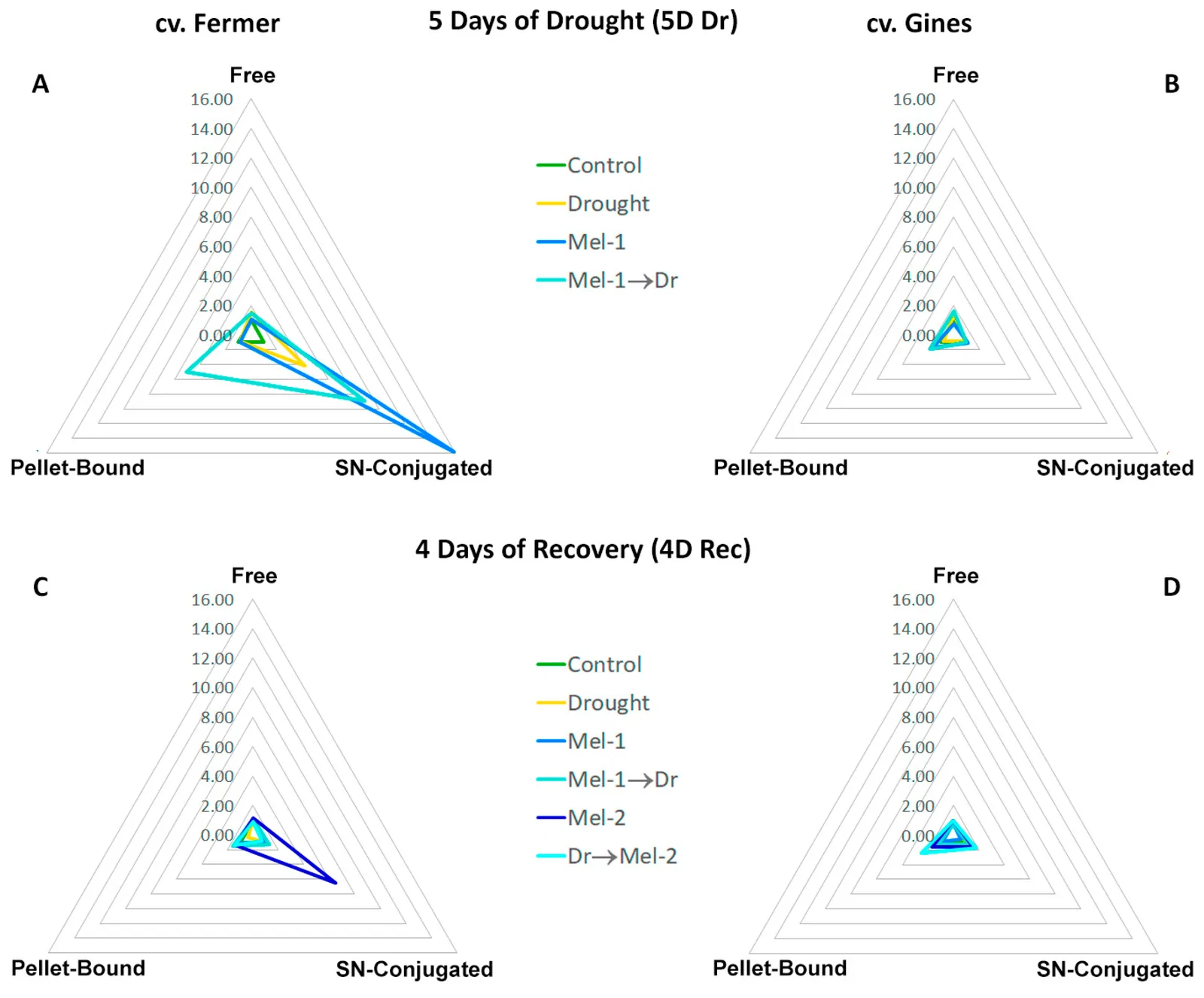
Radar plot of free, SN-conjugated and pellet-bound fractions calculated as sum of measured polyamines (putrescine + spermidine + spermine) at the fifth day of drought (5D Dr (**A**,**B**)) and four days into recovery (4D Rec (**C**,**D**)). The abbreviations within the legends denote the following—Control: normally irrigated non-treated; Drought: 5 days of water deficit; Mel-1: melatonin control before drought; Mel-2: melatonin control after drought; Mel-1→Dr: melatonin pre-treatment before drought; Dr→Mel-2: melatonin post-treatment after drought. The presented values are normalized to the respective control.

The amplitude alterations of the polyamines (Figure 2, Figure 3 and Figure 4), calculated as the sum of their free, SN-conjugated or pellet-bound (putrescine + spermidine + spermine) fractions at 5D Dr (Figure 5A,B) and 4D Rec (Figure 5C,D) presented as radar plots in cv. Fermer (Figure 5A,C) and cv. Gines (Figure 5B,D); they revealed that drought predominantly caused significant deviations from the control levels in the less-drought-tolerant variety, causing induction of SN-conjugated polyamines (5D Dr) and reduction in the pellet-bound fraction (4D Rec). Pre-treatment with melatonin also led to a greater effect during the studied period and to higher accumulation of SN-conjugated polyamines at 5D Dr than in cv. Gines. Similarly, both melatonin treatments applied alone highly induced the sum of the studied SN-conjugated fractions in the less-drought-tolerant variety, while in cv. Gines, the effects of the same melatonin applications were significantly less expressed. Generally, the alterations in cv. Fermer were most pronounced at 5D Dr. When melatonin was applied after the stress was ceased (Dr→Mel-2), it predominantly induced pellet-bound polyamines in cv. Gines.

#### 2.2.5. Principal Component Analysis (PCA)

**Figure 6:**
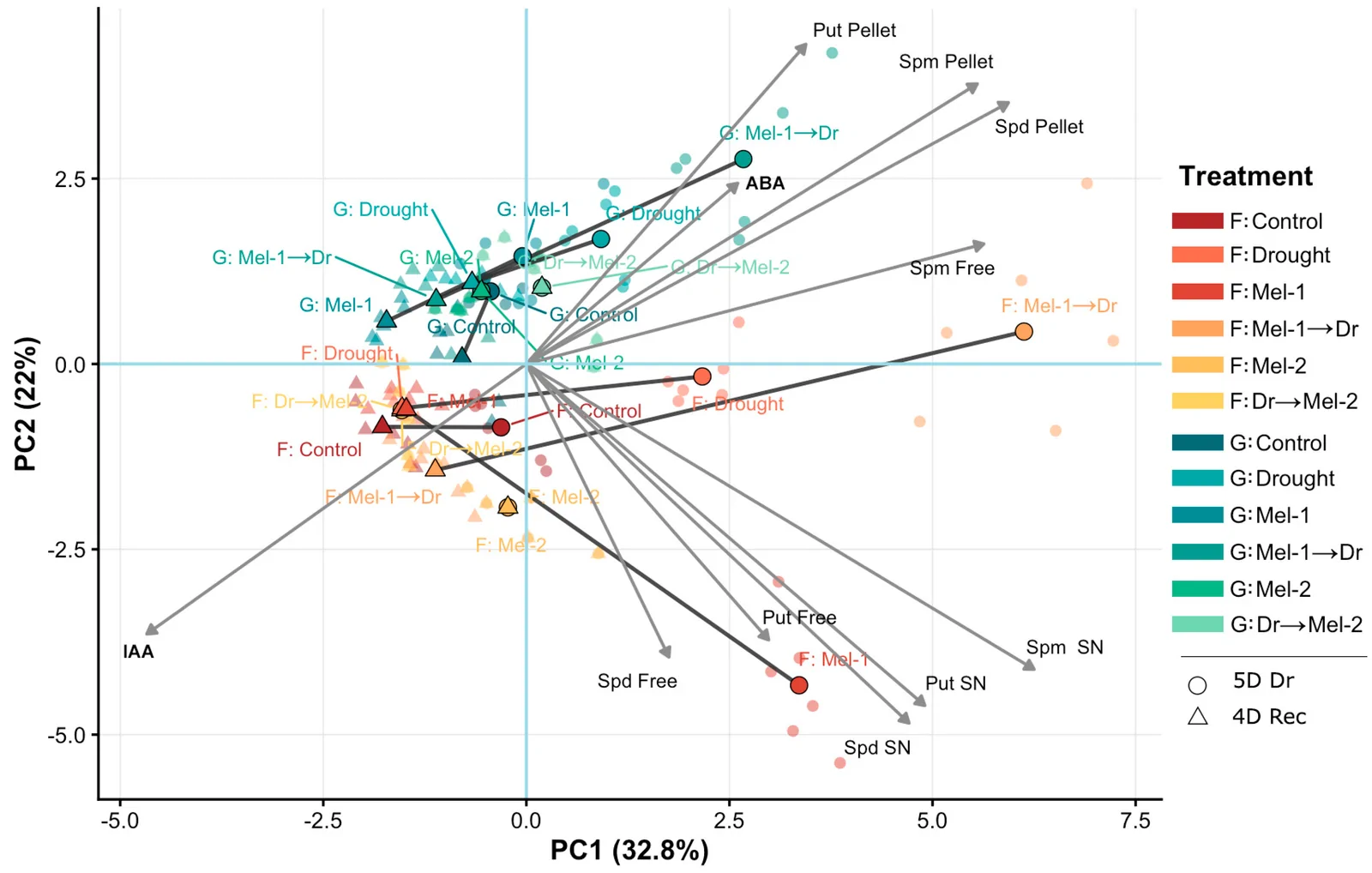
Principal component analysis of IAA, ABA, and PA profile variations after treatment with melatonin, drought and a combination of them in cv. Fermer (F) and cv. Gines (G) at the fifth day of drought (5D Dr, ○) and four days into recovery (4D Rec, △). Abbreviations used correspond to ABA: abscisic acid; IAA: indole-3-acetic acid; Put Free: free putrescine; Spd Free: free spermidine; Spm Free: free spermine; Put SN: SN-conjugated putrescine; Spd SN: SN-conjugated spermidine; Spm SN: SN-conjugated spermine; Put Pellet: pellet-bound putrescine; Spd Pellet: pellet-bound spermidine; Spm Pellet: pellet-bound spermine; Control: normally irrigated non-treated; Drought: 5 days of water deficit; Mel-1: melatonin control before drought; Mel-2: melatonin control after drought; Mel-1→Dr: melatonin pre-treatment before drought; Dr→Mel-2: melatonin post-treatment after drought. The different colors correspond to the different treatments.

The PCA of the 5D Dr and 4D Rec datasets revealed cultivar- and treatment-dependent variation in the hormonal and PA profiles of cultivars Fermer and Gines (Figure 6). PC1 and PC2 explained 32.8% and 22.0% of the total variance, respectively, accounting for 54.8% cumulatively.

The variable vectors indicated that PA partitioning was a major source of variation. Pellet-bound Put, Spd and Spm fractions were oriented toward positive PC2, whereas their SN-conjugated fractions were related predominantly to negative PC2. Free Spm was associated with positive PC1 and PC2, while free Put and free Spd pointed mainly toward negative PC2. IAA was oriented toward negative PC1 and PC2, whereas ABA was associated with positive values of both components. This indicates the contrasting patterns of variation among hormonal and PA parameters.

All treatment groups of cv. Gines, particularly those involving drought, were positioned toward the pellet-bound polyamine vectors. In contrast, in cv. Fermer, Mel-1 treatment showed a stronger association with the SN-conjugated fractions. The displacement of treatment groups between drought and recovery further indicated that metabolic changes depended on cultivars and treatments, rather than reflecting a mechanistic return to the control profile.

Overall, the PCA highlights cultivar-dependent reorganization of PA pools during drought and early post-drought recovery stages, particularly the redistribution of PAs among free, SN-conjugated and pellet-bound fractions. These patterns suggest coordinated changes in PA metabolism and hormonal status.

## 3. Discussion

The positive outcome of melatonin application is acknowledged because of its multilevel effects on antioxidant defense, improvement in redox homeostasis and photosynthesis, osmotic adjustment, and modulation of plant growth regulators, their content and interactions [82]. Exogenous melatonin was found to play an important role in abiotic stress protection. Model drought stress using soil most accurately emulates naturally occurring water shortages compared to that of hydroponic- or agar-based cultures containing non-ionic carbohydrate osmolytes or PEG [75].

The current study continues our previous research [77,78,79] on investigating the effects of melatonin root application before or after soil drought stress on growth, oxidative stress markers (hydrogen peroxide and malondialdehyde content and leakage of electrolytes), proline-related transcripts, osmolytes, photosynthesis and antioxidant defense in two wheat varieties with different tolerances [80,81]. We recently reported that moderate drought induced distinct responses of enzymatic and non-enzymatic antioxidant defense in both wheat cultivars [79]. The outcome of melatonin application is related to attenuation of negative drought-induced consequences on photosynthesis and antioxidant response, selective modulation of the transcript expression profile of antioxidant enzymes, and its effects, which depend on the wheat variety’s potential to cope with stress [77,78,79].

Wheat cultivars differing in drought tolerance were documented to have distinct variety-specific responses to stress and possess different adaptation strategies under water-limited conditions [83]. For example, the application of polyamines was reported to mitigate the effects of PEG stress depending on the genetic resources of wheat by involvement in the signaling pathway and slowly adjusting the degree of protective mechanisms, such as antioxidants, soluble sugars and proline, according to the level of stress in the drought-resistant variety [84]. Research studying the effect of melatonin on drought-sensitive and tolerant varieties of *Malus* plants [40] and wheat [64] showed a clearer protective outcome on the sensitive cultivars grown under PEG treatment. Melatonin root drenching decreased ABA in *Malus* species grown under PEG conditions via selective down-regulation of the ABA synthesis gene *MdNCED3* and up-regulation of catabolic genes (*MdCYP707A1* and *MdCYP707A2*), detoxifying H_2_O_2_ [40]. Li et al. [64] showed that melatonin applied in the hydroponic culture with PEG-6000 improved photosynthetic parameters in the drought-sensitive wheat cultivar, but these improvements were associated with increased root IAA and zeatin riboside levels, and decreased contents of leaf ABA, H_2_O_2_, and aminocyclo-propane-1-carboxylic acid. The authors assumed that the results of melatonin application differ depending on the wheat’s sensitivity to drought, but did not explain the positive role of melatonin in the physiological regulation of drought-tolerant cultivars due to insufficient information.

Our study focuses on the effect of root melatonin application before and after moderate soil drought on the levels of IAA, ABA, and PA fractions in the leaves of two wheat varieties with different levels of drought tolerance. The model system used two types of melatonin application: pre-drought, which corresponds to the priming mode of action, and post-drought application 24 h after the initiation of stress recovery. To our knowledge, such information in the literature is absent regarding wheat, especially concerning post-drought melatonin application. The obtained information will expand our understanding of the modulatory effects of melatonin on wheat subjected to soil drought after stress and during early recovery after re-watering.

Auxins are classical growth-promoting phytohormones, with IAA as the main representative, sharing its precursor tryptophan with melatonin. On the other hand, ABA is a stress hormone, usually induced under drought and other adverse conditions, leading to growth inhibition, ROS induction, and modifying plant water content by controlling stomatal conductance [15,19,21,40,61,64,85]. The literature survey showed that in plants grown under non-stress conditions, melatonin application predominantly did not lead to appreciable changes in the levels of IAA [15,38,86] and ABA [15,19,22,23,40,41,71], and our data is in line with these observations. However, IAA and ABA contents could vary depending on the melatonin treatment approach, plant species, and stage of plant development [19,85,87,88].

A drought-induced decrease in IAA content found in both cultivars is consistent with the previously reported reduction in biomass [77] and with the lower level of IAA under osmotic stress [15,19,38]. The drought-induced increase in ABA levels found in both varieties also corresponds with the previously reported reduction in photosynthetic performance [78] and increased contents of ROS-related stress markers such as malondialdehyde, electrolyte leakage, and H_2_O_2_ [77]. The cultivar-specific drought alterations in IAA content during the early recovery period appear to depend on wheat tolerance to stress, indicating the greater potential of the more drought-tolerant cultivar Gines to cope with water deficiencies and recover its IAA content faster than the less-drought-tolerant variety Fermer. The better recovery of IAA levels probably contributes to the attenuated adverse effects of stress-induced oxidative stress and corresponds to the higher number of antioxidant defense compounds reported in cv. Gines [79]. This assumption is consistent with the positive effect of IAA on the antioxidant defense reported by other authors [17,89]. It could also be speculated that the internal capacity of cv. Gines to preserve its IAA content is higher than that of the less-tolerant Fermer variety after drought stress is removed. In addition, the higher increase in ABA content in the less-drought-tolerant variety correlated well with the reduced photosynthesis in cv. Fermer caused by stomatal limitation under drought stress conditions [78]. However, during early post-drought recovery, the ABA levels were still enhanced in a comparable manner in both wheat varieties, providing evidence that the residual negative effect of stress still lasts four days after re-watering. The obtained data is in line with the earlier reported opposite effects of IAA and ABA under drought stress [15,19]. In addition, PCA (Figure 6) also visualizes the same correlations.

A relationship between ABA signaling and polyamine metabolism was described to contribute to the controlled regulation of Spd and Spm content in wheat plants grown under PEG conditions [61]. An increased content of polyamines is commonly reported due to stress, including drought, in an attempt to decrease stress associated with negative consequences on membranes, macromolecules and ROS induction [54,62,73]. The amplified levels of total Put, Spd and Spm observed in the less-drought-tolerant wheat variety after moderate water shortage are consistent with the above findings. However, under prolonged drought conditions, an increased content of cell-wall-bound (pellet-bound) PAs was reported in triticale on account of the reduction in free PAs [63], and a clear reduction in all PA fractions (except free Spm) was detected in wheat [62]. Our data showed that moderate soil drought induced free fractions of Spm in the studied wheat varieties, and this accumulation probably contributed to better plant defense against drought. This suggestion is in line with the well-known fact that Spm possesses the highest protective capacity because of its greatest number of amino groups and respective positive charge [90]. The results also correspond with the reported induction of free Spm and Spd after PEG treatment in drought-resistant wheat varieties [91]. In addition to the occurrence of polyamines as free molecules, they conjugate with small molecules like phenolic acids (SN-conjugated fraction) or are bound to macromolecules (pellet-bound fraction) such as nucleic acids and proteins [92]. We observed cultivar-specific stimulation of SN-conjugated polyamines in the less-drought-tolerant cv. Fermer and, to some extent, the pellet-bound Spm in cv. Gines after drought. The specific induction of the SN-conjugated fraction probably suggests that the two cultivars employ different strategies for maintaining redox homeostasis under drought: cv. Fermer appears to undergo stronger metabolic reprogramming of polyamine pools, whereas cv. Gines relies more strongly on activation of antioxidant defense. The assumption is consistent with earlier findings regarding a higher number of antioxidant defense participants in cv. Gines than in cv. Fermer [79], as well as the fact that SN-conjugated PAs are known to act as ROS scavengers [93]. The accumulation of SN-conjugated polyamines in cv. Fermer could be associated with improved regulation of oxidative stress response under drought, suggesting that polyamine conjugation may participate in the maintenance of redox homeostasis during stress. Additionally, Du et al. [94] reported that the conjugated polyamines in the root plasma membrane could be involved in membrane stabilization. The significant decrease in pellet-bound PA fractions in cv. Fermer after four days of recovery could indicate that the negative effect of moderate drought stress continues even after four days of re-watering. At the same time, in cv. Gines, the increased PA pellet-bound fraction could suggest that this variety possesses a greater ability to maintain cellular membrane stability.

Our data showed that under drought conditions, melatonin application diminished the induced accumulation of ABA and increased the reduced IAA in both cultivars. Based on experiments with ABA and melatonin loss-of-function Arabidopsis mutants, it was suggested that the functional redundancy of melatonin and ABA effects could contribute to their antagonistic action [95]. The authors showed that this type of relationship between ABA and melatonin may provide increased antioxidant defense and stress resistance. Our results are in line with the previously acknowledged antagonistic relationship between melatonin and ABA, particularly when melatonin was applied before drought stress (Mel-1→Dr) in both wheat varieties. For example, Imran et al. [21,22] showed a similar antagonistic effect of multiple or single application of root-applied melatonin pre-treatment on ABA content and the related metabolic genes in *Glycine max* L. subjected to soil drought [21] or high temperature [22]. An earlier study also reported melatonin’s capability to have an indirect positive impact on enzymatic antioxidant activities in addition to its direct action as a hydrogen peroxide scavenger, contributing to the improvement of stomatal function via re-opening, as well as water balance benefits [40]. The indirect mechanism by which melatonin pre-treatment acts was proposed to include a reduction in ABA biosynthesis and induction of ABA catabolic processes in *Malus* species grown under drought [40]. Therefore, the high reduction in the ABA content at 5D Dr, found after melatonin application before drought (Mel-1→Dr) in the less-drought-tolerant variety, showed that melatonin has a greater protective effect in cv. Fermer than in cv. Gines. The data obtained correspond with the previously reported better pronounced effect of melatonin pre-treatment on the attenuation of the drought-induced antioxidant response [79] and H_2_O_2_ accumulation [77], as well as the better recovery of the net photosynthesis rate [78] in cv. Fermer. A similar, but weaker effect on ABA content was observed after melatonin post-treatment (Dr→Mel-2) in cv. Fermer, probably because of the different physiological states at which the plants received melatonin application. It should be noted that the alteration of the stomatal behavior by melatonin application observed in tomato plants subjected to combined heat and drought stress was not caused by ABA regulation but was rather mediated by the effects on ROS metabolism [72].

The positive effect of melatonin pre-treatment on IAA was also better pronounced in the less-drought-tolerant cv. Fermer. After re-watering, the recovery of IAA content in cv. Fermer by melatonin pre- and post-treatment (Mel-1→Dr and Dr→Mel-2), compared to plants only exposed to stress, might be a cultivar-specific strategy for adaptation. Talaat [19] also reported that melatonin application maintained higher IAA content in barley plants subjected to drought stress. However, based on the current data, a distinct conclusion could not be made as to whether melatonin application influenced the conjugation, degradation, biosynthesis and/or transport processes of endogenous IAA and ABA in the studied wheat cultivars grown under drought conditions.

In our study, when Mel-1 and Mel-2 were applied alone, they induced specifically SN-conjugated PA fractions in the less-tolerant variety. Thus, the conversion between different PA fractions seems to be an important component in melatonin-induced polyamine modifications to aid higher levels of wheat protection after drought stress. Recently, the participation of polyamine fractions in melatonin-induced tolerance was demonstrated in drought-sensitive foxtail millet under drought stress [73], and in rice under salinity [96]. Additionally, a melatonin-mediated induction of major PA biosynthetic enzyme activities (including those of arginine decarboxylase, ornithine decarboxylase and S-adenosyl-L-methionine decarboxylase), and a decrease in the activities of catabolic enzymes (polyamine oxidase and diamine oxidase) were reported in the roots and leaves of foxtail millet after PEG-6000 [73] and in rice seedlings after NaCl [96]. Melatonin raised all measured PA (Put, Spd, and Spm) fractions (free, SN-conjugated and pellet-bound) in foxtail millet [73]. However, in rice exposed to osmotic stress, melatonin induced free Spd and Spm at the expense of free Put, and also increased bound Spd and Spm fractions but decreased conjugated PAs [96]. Unlike the drought-sensitive foxtail millet [73], our study showed variety-specific inductions of different PA fractions in wheat cultivars after Mel-1→Dr. The melatonin application (Mel-1→Dr) induced both SN-conjugated and pellet-bound fractions in cv. Fermer under drought stress. This amplification was particularly observed in Spm and Spd, which could provide better protection in the less-drought-tolerant variety. However, in cv. Gines, a similar but lower induction was observed only in pellet-bound Spm and Spd after Mel-1→Dr. Pellet-bound PAs are often associated with stabilization of the negatively charged macromolecules [97], and our results are in line with this concept. Therefore, the less-drought-tolerant variety Fermer seems to involve both fractions of SN-conjugated and pellet-bound PAs in its melatonin-induced defense mechanism, potentially reducing the accumulation of ROS-related stress markers and enhancing the macromolecule stability of membranes [77]. Ke et al. [38] found that melatonin alleviated osmotic stress by lowering NaCl-induced free polyamine degradation through inhibition of diamine oxidase and polyamine oxidase, while concurrently amplifying the metabolic flux from arginine and methionine to free PAs in wheat. After restoring the normal water regime, the pellet-bound Put, and to some extent, the SN-bound Spm seem to actively participate in the early post-drought recovery processes in cv. Gines caused by melatonin application after stress (Dr→Mel-2). In addition, in cv. Fermer, both types of melatonin application before and after drought (Mel-1→Dr or Dr→Mel-2) restored the stress-depleted pellet-bound polyamine levels and probably stabilized macromolecules.

Generally, the dynamics of PA alterations appear to differ in the studied wheat varieties. The most substantial changes in the less-drought-tolerant cultivar Fermer were detected five days after drought and are markedly connected with the induction of SN-conjugated fractions, and to a lesser extent, are associated with the induction of the pellet-bound fractions after Mel-1→Dr (Figure 5). Later, after four days of recovery, cv. Fermer experienced reduced pellet-bound PA levels under drought only, whereas plants subjected to the combined treatments had enhanced pellet-bound levels, and Mel-2 induced SN-conjugated polyamines.

The principal component analysis (Figure 6) distinguished the multivariate biochemical profiles of the studied cultivars under drought and early post-drought stages of recovery. All treatment groups of cultivar Gines were present in the positive PC2 region and showed stronger association with pellet-associated PA fractions such as pellet-bound Put, Spd and Spm, as well as free Spm. Conversely, the profile of Mel-1 treatment alone of cv. Fermer showed a stronger association with SN-conjugated PA fractions. Interestingly, Mel-1-primed plants subsequently subjected to drought (Mel-1→Drought) in cv. Fermer were particularly located in the positive PC2 region, in the area covered by the treatment groups of cv. Gines. This alteration indicates that pre-treatment with melatonin was related to a significant reorganization of the plant growth regulators measured in cv. Fermer under drought conditions. The pattern of differences among the cultivars in drought tolerance established before [80,81] supports the hypothesis that melatonin pre-treatment triggers a similar effect in cv. Fermer, resembling the one observed in cv. Gines. However, PCA alone does not provide proof that this shift is due to improved tolerance of the less-drought-tolerant variety. Instead, this interpretation is supported by our earlier published data on physiological and growth-related traits [77,78,79]. Overall, the results highlight the cultivar-dependent regulation of polyamine partitioning and suggest that the metabolic alterations associated with melatonin might be involved in the wheat drought response.

The overall data suggest that cv. Fermer responds to drought through melatonin-modulated reorganization and accumulation of polyamines, particularly reflecting their conjugated forms, whereas cv. Gines maintains a comparatively stable polyamine pool with smaller treatment-induced changes. However, based on the current data, we cannot determine whether melatonin directly stimulated polyamine biosynthesis, reduced degradation, promoted conjugation, altered transport, or affected several of these processes simultaneously.

## 4. Materials and Methods

### 4.1. Plant Material, Growth Conditions and Experimental Design

Two Bulgarian winter wheat (*Triticum aestivum* L.) varieties differing in their tolerance to drought [80,81], namely cv. Fermer and cv. Gines, were used and selected at the Institute of Plant Genetic Resources, “K. Malkov”, Sadovo (Bulgaria). The growth conditions and experimental design are already described in detail in [77,78,79]. Plants were grown in pots containing 560 g of soil in a growth chamber under the following conditions: 200 μmol/m^2^/s photon flux density, 14/10 h photoperiod, and 21 °C/19 °C day/night temperatures. Each pot contained 20 plants. Seventeen-day-old plants with fully developed second leaves were subjected to five days of soil drought via watering termination. Plant treatments were divided into six groups as follows:

**Control**—Soil moisture was maintained at 75% of the field soil capacity via daily watering after weighing the pots;

**Mel-1**—24 h before the beginning of the drought stress, some of the control seedlings were root-supplemented with 10 mL of 75 µM melatonin as part of water irrigation;

**Drought (Dr)**—Five days of soil drought were observed (where field soil capacity reached 15% at the end of the stress period);

**Mel-1→Dr**—Some of the pre-treated Mel-1 plants were subjected to drought;

**Mel-2**—24 h after the end of the stress period, some of the control seedlings were root-supplemented with 10 mL of 75 µM melatonin as part of water irrigation;

**Dr→Mel-2**—24 h after the end of the stress period, the parts of the plants subjected to drought were root-supplemented with 10 mL of 75 µM melatonin as part of water irrigation.

Each sample for the biochemical analyses was collected as pooled material derived from the above-ground part of three plants with one pot per sampling point: before drought (0D Dr), at the fifth day of drought (5D Dr), and four days into the recovery period following drought (4D Rec). Each sample was stored at −80 °C until analyses.

### 4.2. Comparative Analyses of Polyamines

Fresh plant material (approx. 0.3 g) was ground with 3 mL of 5% trichloroacetic acid and then centrifuged at 3000× *g* (4 °C) for 15 min. The supernatant was used to determine the free Put, Spd and Spm concentrations via direct dansylation [98]. The conjugated fractions of PAs were measured according to the method of Torrigiani et al. [99]. The acid-hydrolyzed supernatants were used as sources for SN-conjugated PAs, and the acid-hydrolyzed pellets were used for pellet-bound PAs, respectively. The hydrolyzed conjugated and bound PAs were dansylated and further processed following the protocol for measuring free polyamines by thin-layer chromatography (TLC). TLC pre-coated plates of Silicagel G 60 (Merck, Darmstadt, Germany) were used to separate the individual dansylated PAs using a solvent system of cyclohexane:ethylacetate (3:2, *v*/*v*). The spots, visualized under ultraviolet light, were scraped off and eluted in anhydrous acetone. Fluorescence (excitation 360 nm, emission 505.5 nm) was measured on a Shimadzu spectrofluorophotometer RF-1501 (Shimadzu, Kyoto, Japan) and the results were compared with dansylated PA standards obtained from the same TLC plate.

### 4.3. Analyses of IAA and ABA Concentrations

The content of the phytohormones IAA and ABA was assessed by the ELISA method [100]. Approximately 1 g of fresh leaf material was homogenized in 80% methanol and extracted overnight at 4 °C with constant shaking. After centrifugation at 15,000 rpm (30 min) the resultant supernatant was prepared and derivatized using kits for IAA (Cat. Number 010 1531) and ABA (Cat. Number 010 2701) according to the manufacturer’s instructions (OlChemIm Ltd., Olomouc, Czech Republic). The absorption was recorded at 405 nm with a UV-VIS spectrophotometer (Thermo Fisher Inc., Waltham, MA, USA) using SkanIt Software 2.2 (Thermo Fisher Inc.) and concentrations of IAA and ABA were calculated using standard curves.

### 4.4. Statistical Analyses

Two independent experiments with three biological replicates to obtain the data presented for all biochemical assays (*n* = 6). The data represented are average values with standard error (SE). One-way ANOVA with Duncan’s post hoc multiple range test was used to assess the statistical significance of the treatments at the 0.05 level. The PCA was performed in RStudio version 2026.09.0+174 with R version 4.6.1 (Happy hop).

## 5. Conclusions

In conclusion, drought led to specific alterations in IAA, ABA and polyamine levels post-stress and after early post-drought recovery, which corresponds to the tolerance of the studied varieties. After drought stress, the less-tolerant cv. Fermer did not recover the decreased IAA level, in contrast to the drought-tolerant cv. Gines. Correspondingly, the induction of ABA was stronger at the end of the stress period in cv. Fermer than in cv. Gines. The less-drought-tolerant variety showed more pronounced changes in the polyamine pool, indicating a stronger reaction to the applied stress.

The melatonin-induced alterations in IAA, ABA, and polyamines had a positive effect on the endogenous plant growth regulators of the less-tolerant variety of Fermer grown under drought stress. The presented data suggest a complex interaction between the melatonin application and the polyamine pool, which is alleviating the negative effects in the cultivars Gines and Fermer under moderate drought conditions. Consistent with other authors, we confirmed the role of melatonin as a master growth regulator by showing its modulatory effects on IAA, ABA, and the polyamine pool. Melatonin application (before and after drought) stimulated IAA restoration, mitigated drought-induced ABA, and amplified specific polyamine fractions in both cultivars, reflecting their tolerance.

The recovery processes after drought have not been studied enough, despite their importance for ensuring sustainable plant development and adaptive management. Therefore, it is important to study the physiological processes that occur after a longer period of recovery along with the modulatory effects of melatonin not only after moderate but also after severe stress and not only under controlled conditions but also in more realistic field environment.

## Data Availability

The original contributions presented in this study are included in the article. Further inquiries can be directed to the corresponding author.
